# Energy performance of MRI systems: on-site validation and comparison with manufacturer declarations

**DOI:** 10.1186/s41747-025-00668-w

**Published:** 2026-01-05

**Authors:** Andrea Roletto, Matteo Verga, Anna Savio, Gian Luca Viganò, Simone Zanoni

**Affiliations:** 1 Department of Mechanical and Industrial Engineering, Università degli Studi di Brescia, Brescia, Italy; 2 Clinical Engineering Unit, ASST Spedali Civili di Brescia, Brescia, Italy; 3 Department of Electronics, Information and Bioengineering, Politecnico di Milano, Milano, Italy; 4 Department of Civil, Environmental, Architectural Engineering and Mathematics, Università degli Studi di Brescia, Brescia, Italy

**Keywords:** Carbon footprint, Conservation of natural resources, Electricity, Magnetic resonance imaging, Radiology

## Abstract

**Objectives:**

This study aims to evaluate the actual energy consumption of two generations of 1.5-T magnetic resonance imaging (MRI) scanners, quantify the benefits in terms of primary energy savings resulting from technological replacement, and compare field estimates of primary energy consumption with those reported in environmental product declarations (EPDs).

**Materials and methods:**

Two 1.5-T MRI scanner models, the old model version and its new model replacement, were monitored using a power quality analyzer connected to the electrical cabinet. Electrical power consumption data were collected over 2-week periods, both before and after the scanner replacement. Primary energy consumption was projected over 10 years, and the resulting values were compared with those reported in the EPDs for the two scanners.

**Results:**

Over 10 years, cumulative energy consumption is estimated to be 1,010.4 MWh for the new unit *versus* 1,206.7 MWh for the old unit, corresponding to a 16.3% reduction. Considering the range of European primary energy factors (PEFs), energy savings varied from 235.6 to 687.1 MWh. Comparison with EPDs revealed significant discrepancies (± 40%) depending on the national PEF used, demonstrating that EPDs can both overestimate and underestimate actual energy consumption.

**Conclusion:**

Replacement of an old MRI model resulted in measurable energy savings, particularly in non-productive phases. However, EPDs do not always reflect clinical operation or the impact of national energy mixes. While energy efficiency is central to sustainable radiology, it should not be the sole driver for equipment replacement, which must remain primarily guided by clinical and diagnostic criteria.

**Relevance statement:**

For a radiology department focused on more sustainable practices, it is essential to have accurate data on the environmental performance of medical imaging equipment, which should not be based solely on EPDs, but on real data based on usage patterns and national energy mixes.

**Key Points:**

- Replacing the old MRI scanner reduced energy consumption by 16.3%, mainly due to lower use in non-productive modes.
- Over a 10-year operational period, the primary energy consumption savings varied from 235.6 to 687.1 MWh.
- A discrepancy emerged between EPD-reported and real-world measurements, highlighting the importance of on-site validation for sustainability assessments.

**Graphical Abstract:**

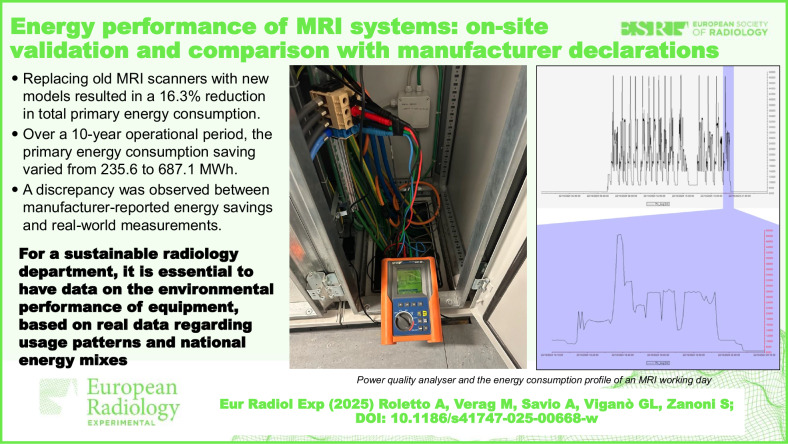

## Background

In recent years, the healthcare sector has paid increasing attention to environmental sustainability, especially in high-energy consumption departments such as radiology [1–5]. Magnetic resonance imaging (MRI) scanners, essential for diagnosing a wide range of medical conditions, are known for their substantial energy requirements, intended as the electrical power required by the MRI scanner to perform daily diagnostic activities [6, 7]. Various studies estimate the annual energy consumption of an MRI scanner to exceed 100,000 kWh [8, 9]. In view of this, several studies have examined the possibility of introducing operational strategies aimed at reducing both energy consumption and the carbon footprint of MRI scanners.

Among the most frequently discussed energy-saving strategies in the literature is the management of the MRI’s power usage across different operating phases [10–12]. Indeed, it has been estimated that simply switching from “idle” mode to “power save” mode during non-operational hours can reduce energy consumption by 25–33%. This mode adjusts hardware components for reduced power use, enabling substantial energy savings [10]. Recent studies have also investigated energy consumption from different perspectives, such as protocol-level determinants and operational management options [13, 14] or broader strategies integrating ecodesign and sustainable operations for MRI and CT [15].

Leading companies in the medical imaging technology sector are also making significant progress in reducing the energy consumption of MRI scanners, equipping the latest systems with an “eco-power mode” that regulates the helium compressor’s operation to limit energy usage [16]. As a result, replacing obsolete MRI systems with more modern counterparts could offer considerable advantages in terms of energy efficiency, both at the hardware and software levels [17]. Initial findings have already been reported by the European Coordination Committee of the Radiological, Electromedical and Healthcare IT Industry (COCIR), which observed a reduction in the daily energy consumption of MRI scanners over the years. Data show a drop from 226 kWh in 2011 to 165 kWh in 2017, attributing this progress to both more efficient scanner design and smarter operating practices.

This suggests that next-generation MRI technologies not only deliver improved diagnostic performance but also align more closely with environmental sustainability goals [18]. Energy consumption is one of the most important environmental characteristics of medical devices, as it directly influences both operational costs and long-term environmental impact. The concept of Cumulative Energy Demand (CED) is increasingly used to assess the environmental performance of imaging equipment. CED accounts for the primary energy consumption required to produce, operate, and dispose of a medical device, including all related transportation [19].

**Fig. 1:**
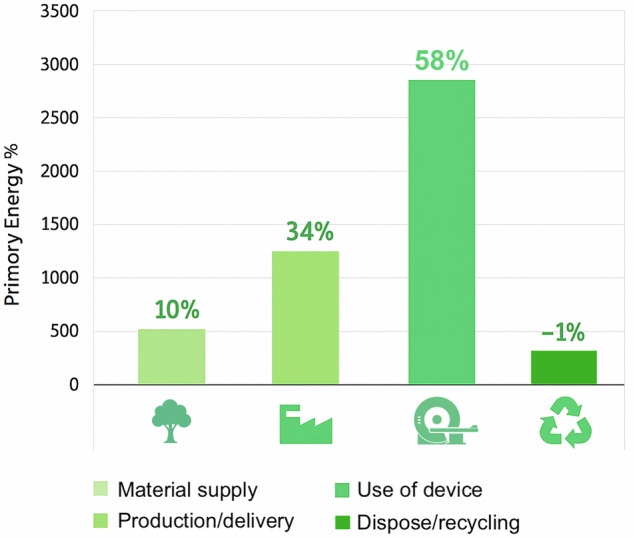
Distribution of primary energy demand across the life cycle stages of an MRI system. The clinical use represents the largest share of total energy consumption (58%), followed by production and delivery (34%), material supply (10%), and end-of-life disposal/recycling, which provides an energy recovery benefit (-1%). These values highlight the predominant impact of operational energy use on the overall environmental performance of the device (Source: siemens-healthineers.com/sola)

Among all these contributing factors, the energy consumed during the clinical use represents the most significant share of the total CED for an MRI scanner throughout its entire life cycle (Fig. 1) [20, 21]. Primary energy consumption includes consumption by the energy sector itself, losses during conversion (*e.g*., from oil or gas to electricity) and energy distribution, as well as final consumption by end users [22]. To derive primary energy consumption from the direct consumption of a device, a primary energy factor (PEF) must be applied for each energy carrier, which varies between countries and depends on the type of energy supply in the country itself [23, 24].

Most available data on the primary energy consumption performance of a radiological device comes from Environmental Product Declarations (EPDs), which are based on standardized assumptions and technical documentation provided by manufacturers. These documents often report significant improvements in energy efficiency in newer MRI models, but such claims require validation through real-world measurements [25]. Alongside this, broader international initiatives have been launched to harmonize sustainability criteria in the procurement of medical imaging equipment. The Medical Equipment Proactive Alliance (MEPA), for example, has developed 32 basic criteria covering energy efficiency, material use and chemical safety, while promoting refurbishment and life extension programs to maximize the useful life of imaging equipment before disposal [26].

At the European level, sustainability-oriented frameworks are also emerging, such as the Green ID Programme from the European Society of Radiology, which aims to support radiology departments in implementing environmentally responsible practices and benchmarking progress across institutions [27]. Such regulatory frameworks emphasize the importance of supplementing manufacturer-reported data with real-world validation, as pursued in this study. However, to date, no studies have directly compared EPD-reported primary energy consumption with actual field data collected in clinical environments.

This study aims to fill a current gap in the literature by providing a comprehensive, field-based evaluation of the actual energy savings achieved when replacing an MRI system with a more efficient model. Specifically, the objective is to measure real-world energy consumption of two MRI scanners, an old unit and its new replacement, during routine clinical use, and to compare these measurements with the values reported in the manufacturers’ EPDs. Based on the verified energy savings, the study also estimates the cumulative primary energy demand over a typical 10-year lifespan of the equipment, converting energy consumption into primary energy equivalents. This allows for an estimation of the potential reduction in environmental impact associated with the replacement.

## Materials and methods

In this study, several data sources were combined to enable detailed data analysis. Two time periods within 2024 were chosen, and the corresponding data were retrieved from each source system. The study was set up to investigate the following aspects: (1) the distribution of energy consumption across different scanner activity states; (2) the differences in energy consumption between two MRI scanners of different generations; (3) the estimation of the cumulative primary energy consumption over a typical 10-year system lifespan; (4) the consistency between energy values declared by the manufacturers in the EPDs and those measured during actual clinical operation.

### Energy consumption measurement of MRI scanners

**Fig. 2:**
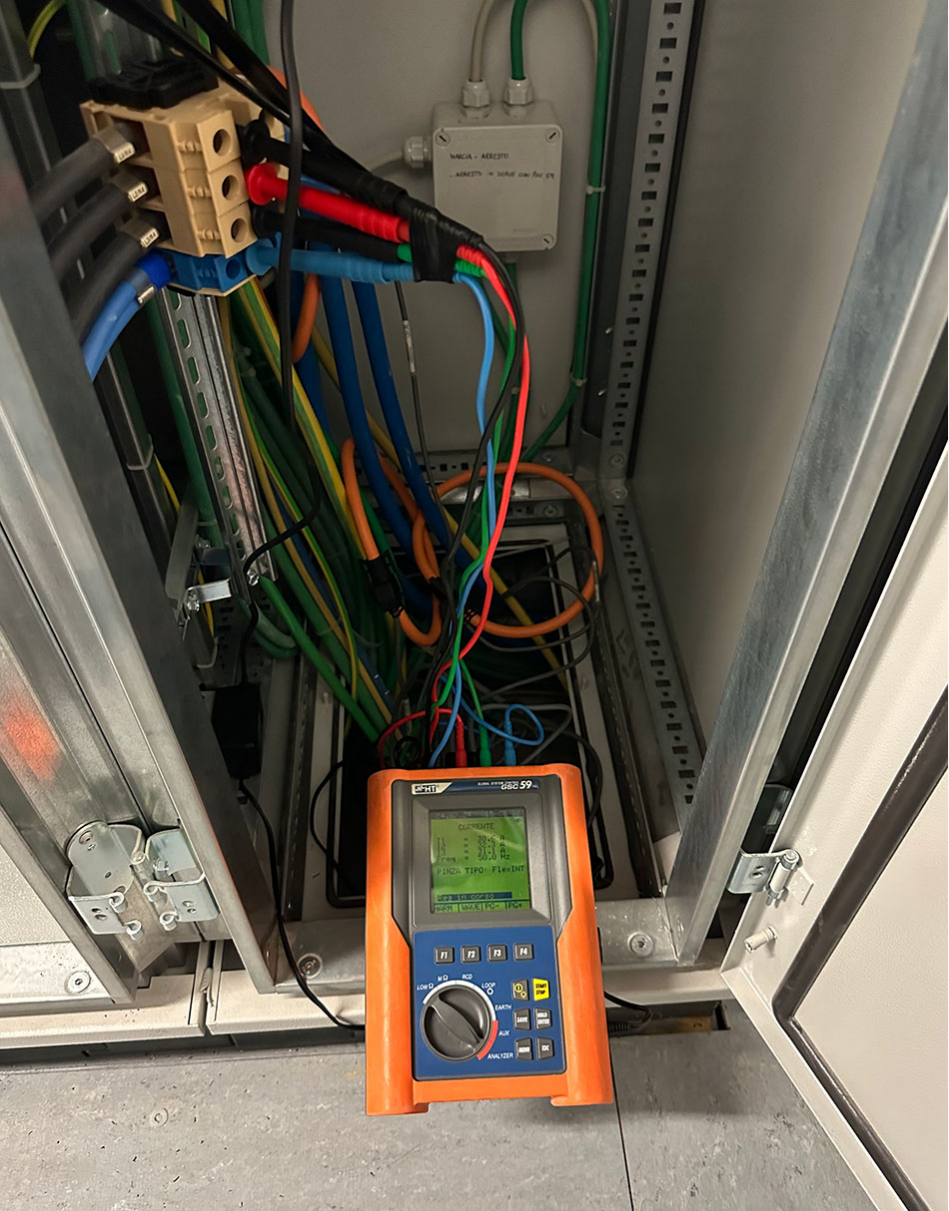
Measurement setup for on-site energy monitoring. Power consumption was measured noninvasively with a power quality analyzer (GSC59, HT Instruments) connected to the incoming power line of the MRI electrical cabinet. The analyzer continuously measured voltage, current and power parameters during routine scanner operation, allowing real-time assessment of energy demand without interfering with clinical workflow

Measurements were performed at the Spedali Civili di Brescia, a 1,294-bed public hospital in the city of Brescia, Italy, in July 2024 in the 2 weeks prior to the decommissioning of an old 1.5-T MRI unit and, in October 2024, in the first 2 weeks of full operation of a new 1.5-T MRI unit. The old unit was a Magnetom Aera (Siemens Healthineers), equipped with software version VA50A, gradient system of 625 A/2,000 V, and a radiofrequency amplifier peak root mean square power of 26.1 kW. The new unit was a Magnetom Sola (Siemens Healthineers) equipped with software version XA61, gradient system of 625 A/2,000 V, and a radiofrequency amplifier peak root mean square power of 29.2 kW. The normal operating hours of the practices are on average between 8 and 12 h from Monday to Friday, occasionally 8 h on Saturdays and only emergency examinations on Sundays. Over the course of 2 weeks, 153 examinations were performed on the Magnetom Aera (old unit) and 180 on the Magnetom Sola (new unit). As regards the energy consumption associated with the type of examinations, a summary is given in the Supplementary Material (Table S1). Both monitoring periods were fully comparable in terms of scanner utilization, operating hours, and activity, as both systems operated during normal opening hours and according to usual scheduling practices. Although the absolute number of examinations differed, the distribution of clinical activity remained representative of standard workflow. The types of examinations and sequences performed were not reported, as they reflect clinical decision-making and protocol optimization beyond the scope of this study. Furthermore, data collection for the Magnetom Sola began after complete installation and configuration with standard clinical protocols, with staff already trained on the system. Power consumption was measured noninvasively with a power quality analyzer (GSC59, HT Instruments), commonly used in hospital infrastructure energy audits (Fig. 2). Measurements of voltage (V), current (A), effective power (W) and frequency (Hz) were carried out on the main supply lines of the MRI scanner, with time recorded. The measurements were analyzed in a 30-s interval. The measured energy consumption (integral of energy consumption over time (kWh)) includes the operation of the MRI scanner, magnet cooling and the reconstruction server, but not external cooling components and auxiliary devices. Radiology information system (RIS) data containing workflow information describing the start and end of the examination were overlaid on the data collected by the power meter to determine the energy consumption associated with the different states of the scanner activity system. All data were collected by the first and senior authors, in collaboration with the Clinical Engineering and Technical Office of the Civil Hospital of Brescia. No vendor involvement or support was provided during the data collection phase, ensuring the independence and neutrality of the study.

### Data analysis

**Fig. 3:**
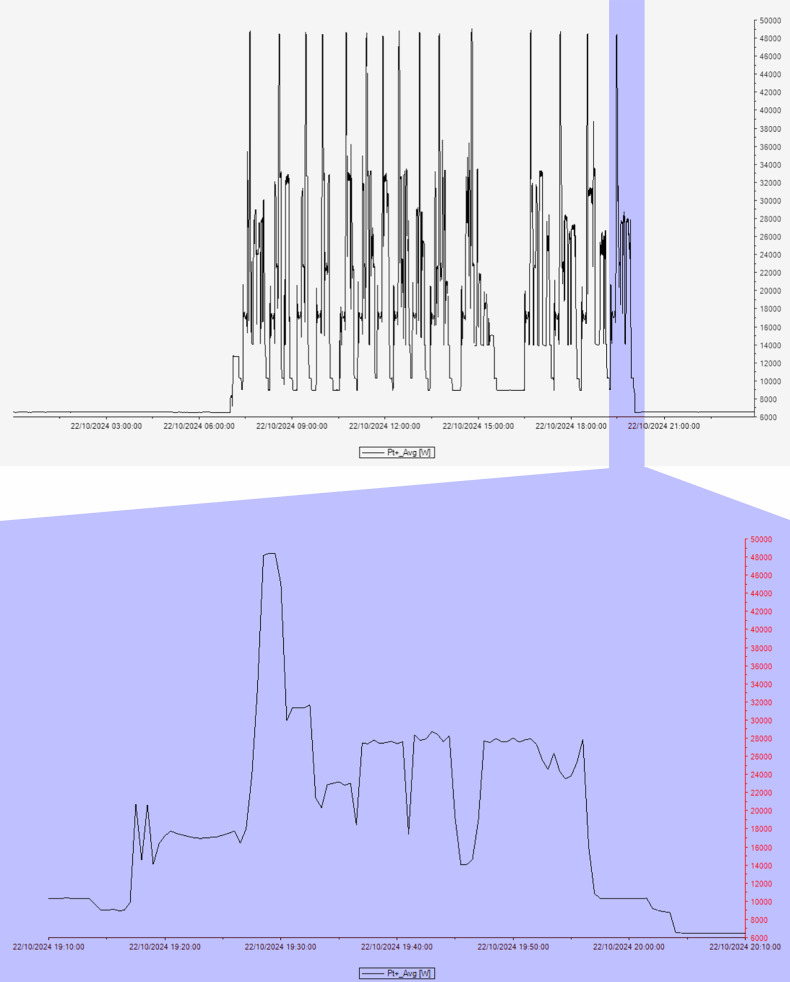
The graphs show the consumption profile (in W) of an MRI unit working day recorded by the power quality analyzer, in which an MRI scan is zoomed in to show the footprint of energy consumption in the active state

Using the data collected by the power quality analyzer, RIS performance data and preliminary analyses performed by the graphical evaluation software for measurement and network data of power analysis (Topview, HT Instruments), energy consumption signals were segmented according to the activity states of the scanners (Fig. 3):

- active state—the actual scanning period of the scanner during which power consumption deviates from the baseline; the production phase in which images are acquired;
- idle state—the time interval between ‘active’ time periods within the system’s power-on period; this is simply a time interval defined within the system’s power-on state and not a production state that can be manually activated.
- stand-by state—the system state in which most components are switched off but can still consume energy due to components that must necessarily remain always switched on (*e.g*., helium compressor pumps); immediate scanning is not possible, and a power-up sequence of several minutes is required before scanning.

### Assessment of environmental product declarations

To better understand how accurately the data provided by manufacturers reflect actual energy requirements, the difference in primary energy consumption measured in the field was compared with that reported in the EPDs for the Magnetom Aera [20] and Magnetom Sola [21] models. These documents, developed according to international standards, provide estimates of primary energy consumption that include all aspects related to the use and maintenance of the equipment, including the energy consumption of heating and cooling systems, based on factory conditions. According to the EPDs, the cumulative primary energy consumption attributable to the use of the devices over 10 years amounts to 3,680 MWh for the Magnetom Aera and 2,874 MWh for the Magnetom Sola, resulting in a difference of -22% between the two generations. For each scanner, energy values were extracted from the respective EPDs, and the difference between the two MRI generations was compared with the difference in primary energy consumption measured in the field during the monitoring period. Unfortunately, EPDs do not indicate which PEF was used to calculate the primary energy consumption of the equipment, so it was not possible to make a direct comparison between the vendors’ measurements and field measurements.

### Estimation of cumulative primary energy demand

To estimate the primary energy consumption of the two scanners of different generations through on-site measurements, the cumulative primary energy requirement was estimated over a 10-year operating period, in accordance with the EPDs released by the vendors, which represents the average lifespan of an MRI scanner in a clinical setting. The cumulative energy requirement was calculated by multiplying the average weekly energy consumption by the number of weeks in a year (52 weeks) and then by 10 years. To convert this value into primary energy, various PEFs for domestic electricity were considered. The European PEF of 1.9, established in the European Union Directive 2023/807 [28], was used, as well as the PEFs for electricity provided by individual EU members. Reported values range from a minimum of 1.2 to a maximum of 3.5, with an average of 2.3 [29].

## Results

### Old MRI unit

In the last 2 weeks of its decommissioning, a total of 153 diagnostic examinations were performed with the 1.5-T Magnetom Aera. For 1 day, clinical activity was suspended to perform quality checks by the department of medical physics (not included in the energy calculations). The total energy consumption for a 2-week period of activity was 4,309.6 kWh. The peak power consumption of the scanner, analyzed per aggregated daily maxima, was 48.7 kW ± 13.4 (mean ± standard deviation), ranging 7.3–60.5 kW, while the minimum power measured was 11.8 ± 0.0 kW during idle state and 7.3 ± 0.0 kW during stand-by state. The average daily energy consumption for the scanner was 307.8 kWh. In total, over the 2 weeks of the observation, the scanner consumed 2,284.9 kWh during the active state, 633.5 kWh during the idle state, and 1,391.2 kWh during the stand-by state (Table 1). Stand-by state accounted for 32% of the total energy consumption owing to the continuous operation of the cold head cooling system. MRI idle state energy consumption accounted for 15% of the total energy consumption.

**Table 1:** Distribution of MRI energy consumption and power according to the type of activity states of two 1.5-T MRI scanners, with additional technical and operational characteristics

Model	Characteristics	Energy consumption (kWh)							Power (kW)					
		Active state	Idle state	Stand-by state	Total	Daily			Power peak demand				Idle state minimum power	Stand-by state minimum power
						Median	Mean	SD	Maximum	Median	Mean	SD		
Magnetom Aera	Software: VA50A Gradient: 625 A/2,000 V RF amplifier: 26.1 kW Commercial release: 2010 Installed at hospital: 2013	2,284.9	633.5	1,391.2	4,309.6	321.0	307.8	66.1	60.5	52.3	48.7	13.4	11.8	7.3
Magnetom Sola	Software: XA61 Gradient: 625 A/2,000 V RF amplifier: 29.2 kW Commercial release: 2018 Installed at hospital: 2024	2,243.1	384.1	1,258.8	3,886.0	293.4	277.6	52.1	54.4	49.0	45.0	13.0	9.5	6.5
**Comparison**	**-2%**	**-39%**	**-10%**	**-10%**	**-9%**	**-10%**	**-21%**	**-10%**	**-6%**	**-8%**	**-3%**	**-19%**	**-11%**	

### New MRI unit

In the first 2 weeks of its installation, a total of 180 MRI examinations were performed with the 1.5-T Magnetom Sola. The total energy consumption for a 2-week period of activity was 3,886.0 kWh. The peak power consumption of the scanner, analyzed per aggregated daily maxima, was 47.8 kW ± 7.7, ranging 29.6–54.4 kW, while the minimum power measured was 9.5 kW ± 0.8 during idle state and 6.5 kW ± 0 during stand-by state. The average daily energy consumption for the scanner was 277.6 kWh. In total, over the 2 weeks of the observation, the scanner consumed 2,243.1 kWh during the active state, 384.1 kWh during the idle state, and 1,258.8 kWh during the stand-by state (Table 1). Stand-by state accounted for 32% of the total energy consumption owing to the continuous operation of the cold head cooling system. Idle state energy consumption accounted for 10% of the total energy consumption.

### Comparison between different activity states of two models

The comparison between the two MRI units is summarized in Table 1. Overall, during different operating states, the reduction is -2% in the active phase, -39% in the idle phase, and -10% in the stand-by phase. Similarly, when looking at average daily consumption, there is a -10% decrease between the two MRI units. Considering power, the average power peak demand decreases by -8% (from 48.7 kW to 45.0 kW) between the old and new models, and the maximum peak power demand is reduced between the old and updated models by -10% (from 60.5 kW to 54.4 kW). The minimum power in waiting mode decreases by -19% and in stand-by mode by -11%.

Regarding the various types of neurological diagnostic examinations performed, Supplementary Table S1 summarizes the comparison between the two scanners. The total number of examinations and their distribution across the different types are similar and comparable. In almost all categories, transitioning from the old model MRI to the upgraded one led to a reduction in average energy consumption, ranging from -3 to -60%. For instance, for noncontrast brain MRI, the average energy consumption decreased from 15.6 kWh with the old MRI unit to 11.8 kWh with the new MRI unit (-32%). As for examination times, the differences between the two models vary according to the type of examination. On average, examinations show a reduction in duration, apart from noncontrast thoracic, lumbosacral and whole-spine, which show an increase.

### 10-year primary energy demands

Based on the weekly electricity consumption observed during the monitoring period, cumulative energy savings over a 10-year system lifespan were estimated. Assuming 52 weeks of operation per year, this corresponds to a total electricity consumption of 1,206.7 MWh for the old MRI unit and 1,010.4 MWh for the new unit. However, to account for the presence of a routine physical quality check interval during the monitoring of the old MRI unit, the extrapolation was adjusted using an effective 13-day activity window for the old scanner and a 14-day window for the new scanner. To obtain the cumulative primary energy consumption, different PEFs applicable across Europe were considered. By applying a reference PEF of 1.9, the cumulative primary energy demand over 10 years amounts to 2,292.7.0 MWh for the old unit and 1,919.7 MWh for the new unit. When applying the individual national PEFs reported across EU member states, the 10-year primary energy consumption for the old unit ranges from 1,448.0 MWh (minimum) to 4,223.4 MWh (maximum), with an average value of 2,775.4 MWh. For the new model 10-year primary energy consumption ranges from 1,212.4 MWh (minimum) to 3,536.3 MWh (maximum), with an average value of 2,323.8 MWh. This would result in a primary energy savings of 16.3%, ranging from 235.6 MWh (minimum) to 687.1 MWh (maximum), with an average value of 436.8 MWh.

### Comparison of primary energy demand with published EPDs

To further contextualize the results, Table 2 compares the 10-year primary energy demand calculated based on on-site measurements using various European PEFs with the values reported in the EPDs. Depending on the national PEF applied, the average difference between EPD values and field-based estimates ranges from -37.7% for the Magnetom Aera to -33.2% for the Magnetom Sola, using the standard EU PEF of 1.9. When applying higher national PEFs, such as 3.5, the calculated demand for the Sola model slightly exceeded the value reported in the EPD (+23.0%), while the Aera model remained broadly in line (+14.8%). These results suggest that EPDs may overestimate primary energy consumption under typical clinical operating conditions.

**Table 2:** Comparison of 10-year cumulative primary energy demand (in MWh) for two MRI scanner models (Magnetom Aera and Magnetom Sola) based on on-site measured electricity consumption and converted using different primary energy factors (PEFs)

Model	Source	PEF	10-year primary energy demand (MWh)	Difference from EPD (%)
Magnetom Aera	EPD	‒	3,680.0	Reference (100%)
	Field data	1.20 (minimum)	1,448.0	-60.7%
	Field data	1.90 (European)	2,292.7	-37.7%
	Field data	2.30 (average)	2,775.4	-24.6%
	Field data	3.50 (maximum)	4,223.4	+14.8%
Magnetom Sola	EPD	‒	2,874.0	Reference (100%)
	Field data	1.20 (minimum)	1,212.4	-57.8%
	Field data	1.90 (European)	1,919.7	-33.2%
	Field data	2.30 (average)	2,323.8	-19.2%
	Field data	3.50 (maximum)	3,536.3	+23.0%

Values are compared with those reported in the respective environmental product declarations (EPDs). The percentage difference indicates the deviation of the field-based estimates from the EPD-reported values

## Discussion

### Energy efficiency gains through technological replacement

The results of this study confirm that replacing an older-generation MRI scanner with a newer model can lead to a meaningful reduction in energy consumption, especially during periods when the system is powered on but not actively scanning. Overall, electricity use dropped by around 10%, with the most substantial savings observed during idle and stand-by states. Active scanning showed only a modest improvement, suggesting that gains in efficiency come less from what happens during image acquisition and more from how the system behaves in between scans. This aligns with what has been suggested in previous studies: smarter management of the MRI’s operational states, such as through eco-modes and automated power-saving functions, can play a major role in reducing energy use [10, 11]. Interestingly, even with updated hardware, stand-by mode still accounted for about 32% of total energy consumption. That points to the persistent energy demands of helium-based cooling systems, which remain active even when the scanner is not in use [30].

One promising development in this area is the adoption of helium-free MRI systems, which could potentially eliminate the need for continuous cryogenic cooling. These newer systems have the potential to significantly reduce baseline consumption, particularly in facilities where scanners are left powered on 24/7 [31–33]. Moreover, the upgrade of the MRI scanner has led to a reduction of -6.1 kW in the peak power demand. This figure appears highly relevant as this reduction can lead to a decrease in demand on the electrical grid, improving its reliability and contributing to overall cost reductions [34–36]. Moreover, when considering the cumulative primary energy consumption over a 10-year period, the differences in energy demand between the old and new MRI models range from a minimum of 235.6 MWh to a maximum of 687.1 MWh, depending on the PEF applied. This variation highlights how national electricity generation mixes can significantly influence the environmental benefits associated with upgrading medical imaging equipment.

### Comparison with environmental product declarations

One of the most relevant contributions of this study is the direct comparison between real-world energy measurements and the figures reported in the manufacturers’ EPDs. When applying the standard EU PEF of 1.9, the primary energy demand measured in the field turned out to be significantly lower than the values declared in the EPDs, by 37.7% for the Magnetom Aera and 33.2% for the Magnetom Sola. Interestingly, when applying the highest national PEF currently reported in Europe (3.5), the picture changes: for the Magnetom Sola, the field-based estimate exceeded the value reported in the EPD by 23%. These differences remained substantial even when using different national PEFs, suggesting difficult adaptability to real practice in EPDs, as confirmed by literature in other fields [25, 37, 38]. Although EPDs are built on standardized methodologies and provide a valuable framework for comparing products, they often rely on assumptions that do not always reflect what happens in practice. Usage patterns, climate conditions, and hospital operating schedules vary widely across facilities.

Another limitation is that EPDs do not always specify which PEF was used in their calculations, which makes it harder to reproduce or verify the data. Finally, the PEF itself is not a fixed value; it varies a lot from one country to another, depending on how electricity is produced [29]. In countries with a greener energy mix, the same MRI scanner could have a much lower environmental impact than in countries that rely more heavily on fossil fuels. This means that, in this case, applying country-specific PEFs is essential to understanding the real impact of MRI systems. Only by combining field measurements with localized energy conversion factors can we gain a realistic picture of how much energy is involved in operating this kind of equipment in healthcare. It should also be noted that on-site energy consumption remains the most relevant parameter for hospitals, as it directly reflects operating costs and local sustainability performance. The conversion to primary energy equivalents using the European Commission’s PEFs was carried out primarily to enable comparison with the values reported in the EPD, which are expressed in terms of primary energy. As the exact PEFs applied by the supplier are not disclosed and may vary over time with changes in European legislation, these results should not be interpreted as absolute data, but rather as a sensitivity analysis. This uncertainty highlights the importance of greater transparency in EPDs and the need to supplement the data declared by manufacturers with real-world validation.

### Implications for sustainable radiology

The results of this study demonstrate that radiology departments aiming to reduce energy consumption should consider the energy performance of MRI scanners as a key decision-making criterion when planning system upgrades. In this context, field energy measurements can be a valuable complement to existing tools, such as EPDs, providing a more accurate picture of actual performance. In recent years, vendors have begun to address sustainability more proactively, as demonstrated by initiatives such as those led by COCIR [18] and the ongoing development of a new Energy Star specification for medical imaging equipment by the U.S. Environmental Protection Agency [39].

By integrating environmental sustainability criteria into competitive procurement processes, radiology departments can move toward more sustainable practices without compromising diagnostic capabilities [40]. In addition to the energy consumption of the equipment, the environmental sustainability of magnetic resonance imaging also depends on auxiliary systems (data storage and “heating, ventilation, and air conditioning” (HVAC) systems) [41, 42], procedure-related waste (*e.g*., contrast media) [43–45], and the behavior and training of radiology staff [46, 47]. Addressing these aspects jointly can lead to greater benefits in terms of system sustainability. However, it is important to note that energy efficiency alone cannot justify the replacement of medical equipment. Decisions to upgrade or replace imaging systems should be based primarily on clinical and diagnostic performance, including image quality, patient flow, safety, and compatibility with evolving clinical protocols. These parameters were not evaluated in the present study, which focused exclusively on energy consumption and sustainability parameters. Therefore, the results should be interpreted as a contribution to the broader discussion on sustainable radiology and not as a stand-alone argument in favor of system replacement.

### Study limitations

This study has limitations that should be considered when interpreting the results.

First, the monitoring period was limited to 2 weeks for each scanner. While this timeframe reflects the department’s average workload, it does not account for potential seasonal variations, public holidays, or unusual scheduling patterns that could impact clinical activity and energy consumption. For the old MRI unit, one of the recorded days contains a routine physical quality check interval without clinical examinations (not included in the energy calculations); therefore, the extrapolation to 10 years was calculated using an effective 13-day monitoring window to ensure proportional accuracy. However, comparable studies have demonstrated that shorter monitoring durations can still yield meaningful insights into equipment performance [48, 49]. We acknowledge that MRI workflow may vary over longer periods due to case mix, scheduling patterns, and operational factors. As such, while the chosen observation window is representative of standard practice within our institution, longer or seasonally distributed monitoring periods would provide a more generalizable estimate of MRI energy performance across different settings.

Second, all measurements were conducted at a single hospital, under specific environmental, operational, and organizational conditions. In addition, both scanners were produced by the same vendor, meaning that our findings reflect a single-center, single-vendor experience. This may further limit the generalizability of the results to other settings, particularly in facilities with different usage profiles, technical infrastructures, vendors, or electricity pricing schemes. The monitoring system employed is not certified as a clinical-grade diagnostic device, which may limit the precision of the measurements when compared to certified metrological instruments.

Lastly, given its primarily technical and engineering-oriented focus, the study did not evaluate clinical parameters, such as image quality, diagnostic efficiency, or patient throughput. While these aspects may not directly affect power consumption, they are crucial for understanding the broader efficiency and sustainability of the system in day-to-day practice. Finally, although extrapolating our results over a 10-year system life cycle introduces assumptions, this approach was adopted to provide a realistic benchmark of cumulative performance and is consistent with previous sustainability studies in radiology [50].

In conclusion, this study provides a real-world assessment of the energy performance of two generations of MRI scanners, highlighting the tangible benefits of technological upgrades in terms of energy efficiency. Replacing an outdated 1.5-T MRI scanner with a more recent model led to an overall reduction of approximately 16% in lifetime electricity consumption, with the most significant savings observed during non-productive phases such as idle and stand-by phases.

A key finding of this work is the discrepancy between the primary energy consumption values reported in the EPDs and field measurements. which reached up to ± 40% depending on the applied Primary Energy Factor. This finding suggests that, while EPDs offer useful standardized information, they may not fully reflect real-world performance. On-site validation should therefore be considered an essential step to ensure reliable sustainability assessments and to inform procurement strategies.

Finally, while energy efficiency is a key component of sustainable radiology, it cannot be the sole criterion for equipment replacement. The findings of this study should therefore be interpreted as a contribution to the broader discussion on sustainability in radiology, rather than as a stand-alone argument in favor of scanner replacement.

## Supplementary information

**Additional file 1:** **Table S1.** MRI Examination Energy Consumption according to type of MRI examination. The green cells identify the reductions in energy consumption and time achieved by the MRI system upgrade. MRA: Magnetic resonance angiography. Data for the old MRI unit were collected over a 13-day interval, while data for the new MRI unit were collected over a 14-day interval.

## Data Availability

Data are available from the authors upon reasonable request.

## Abbreviations

CED: Cumulative energy demand
COCIR: Coordination Committee of the Radiological, Electromedical and Healthcare IT Industry
EPDs: Environmental product declarations
MRI: Magnetic resonance imaging
PEF: Primary energy actor
RIS: Radiology information system
