# Supplementary material for: Energy performance of MRI systems: on-site validation and comparison with manufacturer declarations
Source: Eur Radiol Exp. 2026 Jan 5;10:3. doi: 10.1186/s41747-025-00668-w (PMC12770147; doi:10.1186/s41747-025-00668-w)
Supplement: Supplementary file 1 — Additional file 1: Table S1. MRI Examination Energy Consumption according to type of MRI examination. The green cells identify the reductions in energy consumption and time achieved by the MRI system upgrade. MRA: Magnetic resonance angiography. Data for the old MRI unit were collected over a 13-day interval, while data for the new MRI unit were collected over a 14-day interval. [file 41747_2025_668_MOESM1_ESM.docx]

**Table S1** MRI Examination Energy Consumption according to type of MRI examination. The green cells identify the reductions in energy consumption and time achieved by the MRI system upgrade. MRA: Magnetic resonance angiography. Data for the old MRI unit were collected over a 13-day interval, while data for the new MRI unit were collected over a 14-day interval.

|  | **1.5T MRI scanner Magnetom Aera** | | | | | | | **1.5T MRI scanner Magnetom Sola** | | | | | | | **Comparison** | | | | | |
| --- | --- | --- | --- | --- | --- | --- | --- | --- | --- | --- | --- | --- | --- | --- | --- | --- | --- | --- | --- | --- |
| **Type of MRI examination** | n. exams | Energy Consumption (kWh) | | | Time (min) | | | n. exams | Energy Consumption (kWh) | | | Time (min) | | | Energy Consumption (kWh) | | | Time (min) | | |
|  |  | Mean | Median | SD | Mean | Median | SD |  | Mean | Median | SD | Mean | Median | SD | Mean | Median | SD | Mean | Median | SD |
| **Non-contrast brain** | 32 | 15.6 | 11.5 | 4.3 | 28.1 | 27.0 | 9.7 | 41 | 11.8 | 11.0 | 3.8 | 24.6 | 24.0 | 8.2 | **-32%** | **-4%** | **-14%** | **-14%** | **-12%** | **-17%** |
| **Non-contrast brain and cervical spine** | 18 | 12.6 | 11.1 | 3.9 | 29.6 | 26.8 | 8.2 | 17 | 12.0 | 10.4 | 5.4 | 27.7 | 26.5 | 15.3 | **-6%** | **-6%** | **28%** | **-7%** | **-1%** | **46%** |
| **Non-contrast cervical spine** | 7 | 10.3 | 10.0 | 1.8 | 27.4 | 26.5 | 6.8 | 9 | 8.9 | 8.6 | 1.7 | 24.9 | 24.9 | 5.3 | **-16%** | **-16%** | **-7%** | **-10%** | **-6%** | **-28%** |
| **Non-contrast thoracic and lumbosacral spine** | 5 | 13.5 | 13.8 | 2.4 | 31.7 | 34.5 | 6.0 | 8 | 13.1 | 12.5 | 4.7 | 36.2 | 36.2 | 11.5 | **-3%** | **-10%** | **49%** | **12%** | **5%** | **48%** |
| **Non-contrast lumbosacral spine** | 18 | 9.2 | 9.2 | 1.2 | 20.3 | 20.3 | 2.8 | 23 | 7.7 | 7.0 | 1.6 | 21.8 | 21.8 | 4.4 | **-19%** | **-31%** | **27%** | **7%** | **7%** | **38%** |
| **Non-contrast whole-spine** | 10 | 16.7 | 15.8 | 3.1 | 38.4 | 36.8 | 6.4 | 12 | 14.1 | 13.6 | 4.7 | 39.1 | 39.1 | 12.9 | **-19%** | **-16%** | **35%** | **2%** | **6%** | **51%** |
| **Intracranial and supra-aortic trunk MRA** | n/a | n/a | n/a | n/a | n/a | n/a | n/a | 3 | 24.0 | 20.3 | 10.6 | 64.5 | 50.5 | 26.4 | n/a | n/a | n/a | n/a | n/a | n/a |
| **Intracranial MRA** | 6 | 8.4 | 6.4 | 3.7 | 17.3 | 13.5 | 8.1 | 4 | 6.3 | 5.3 | 2.2 | 14.8 | 12.3 | 5.2 | **-33%** | **-21%** | **-69%** | **-18%** | **-10%** | **-58%** |
| **Contrast-enhanced Brain** | 48 | 16.9 | 14.3 | 3.1 | 32.2 | 32.5 | 6.6 | 53 | 13.6 | 13.0 | 4.8 | 31.3 | 28.0 | 9.9 | **-24%** | **-10%** | **36%** | **-3%** | **-16%** | **33%** |
| **Contrast-enhanced brain and whole-spine** | 3 | 28.3 | 27.3 | 1.9 | 68.0 | 70.0 | 4.8 | 5 | 25.6 | 27.9 | 6.0 | 68.1 | 72.0 | 15.6 | **-11%** | **2%** | **69%** | **0%** | **3%** | **69%** |
| **Contrast-enhanced orbital** | 1 | 16.8 | 16.8 | n/a | 38.0 | 38.0 | n/a | 1 | 17.6 | 17.6 | n/a | 44.0 | 44.0 | n/a | **5%** | **5%** | n/a | **14%** | **14%** | n/a |
| **MR myelography** | 5 | 24.4 | 24.9 | 5.3 | 55.3 | 59.0 | 10.8 | 4 | 14.7 | 16.0 | 4.1 | 44.9 | 44.9 | 11.6 | **-66%** | **-56%** | **-30%** | **-23%** | **-31%** | **7%** |
| **TOTAL** | **153** | **2284.9** | **-** | **-** | **4695.5** | **-** | **-** | **180** | **2243.1** | **-** | **-** | **5249.7** | **-** | **-** |  |  |  |  |  |  |
